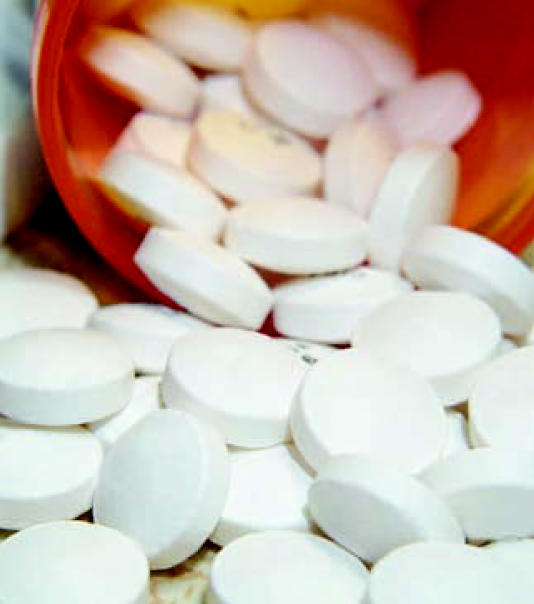# A Twist in the Ritalin Riddle: Drug-Related Genomic Damage Not Confirmed in Children

**Published:** 2007-06

**Authors:** Victoria McGovern

The frequently prescribed central nervous system stimulant methylphenidate (MPH), better known by brand names that include Ritalin, does not cause genomic damage in children, contrary to earlier reports, according to new work published this month **[*EHP* 115:936–940; Walitza et al.]**. In use for more than 50 years and now prescribed more than 5 million times a year in the United States, MPH is the drug of choice for attention deficit/hyperactivity disorder (ADHD). ADHD is the most frequently diagnosed psychiatric disorder in children and adolescents, with an estimated 6–12% of minors worldwide thus diagnosed.

A 2005 report published in *Cancer Letters* had showed that gross genomic damage—reflected by chromosome aberrations including sister chromatid exchanges and formation of micronuclei (smaller-than-normal cell nuclei containing partial genomes)—was found in nucleated lymphocytes taken from peripheral circulation of children who had been taking the drug for only three months. Because large chromosomal breaks are associated with cancer, the study raised concerns about the potential for cancer risk in the millions of people who have taken the stimulant.

That 2005 study found an increased frequency of chromosomal abnormalities in all of the 12 children whose lymphocytes were examined, lending urgency to future studies. The current study looked at micronuclei as an indicator of genomic damage in the lymphocytes of 38 children newly prescribed the drug, following some but not all of them out to six months.

The children, 29 boys and 9 girls, took a variety of doses and formulations of the drug. Some subjects were lost to follow-up during the study; others switched to other medications or dropped out because they did not respond to the drug. Eight children stayed in the study through the whole six months.

Overall, there was no significant increase in the formation of micronuclei at any time point, though some individual children had elevated numbers of micronucleated lymphocytes at one time point or another. Further, the lymphocytes of 9 children who had been taking the drug for more than six months at the start of the study did not show increased levels of micronucleation compared to the pretreatment levels seen in drug-naïve children.

The marked difference in results between the 2005 study and the current one raises the possibility of unexplained genetic differences between the study populations. Whereas the first study population included six white, four black, and two Hispanic children, the latter study focused on a more uniform group of ethnically German children. The authors say further work, especially on the long-terms effects of MPH, is called for.

## Figures and Tables

**Figure:**